# AdipoR agonist increases insulin sensitivity and exercise endurance in AdipoR-humanized mice

**DOI:** 10.1038/s42003-020-01579-9

**Published:** 2021-01-08

**Authors:** Masato Iwabu, Miki Okada-Iwabu, Hiroaki Tanabe, Nozomi Ohuchi, Keiko Miyata, Toshiko Kobori, Sara Odawara, Yuri Kadowaki, Shigeyuki Yokoyama, Toshimasa Yamauchi, Takashi Kadowaki

**Affiliations:** 1 Department of Diabetes and Metabolic Diseases, Graduate School of Medicine, The University of Tokyo, 7-3-1 Hongo, Bunkyo-ku, Tokyo, 113-0033 Japan; 2 PRESTO, Japan Science and Technology Agency, 4-1-8 Honcho, Kawaguchi, Saitama, 332-0012 Japan; 3 Laboratory for Advanced Research on Pathophysiology of Metabolic Diseases, The University of Tokyo, 7-3-1 Hongo, Bunkyo-ku, Tokyo, 113-0033 Japan; 4 RIKEN Structural Biology Laboratory, 1-7-22 Suehiro-cho, Tsurumi-ku, Yokohama, 230-0045 Japan; 5 RIKEN Cluster for Science, Technology and Innovation Hub, 1-7-22 Suehiro-cho, Tsurumi-ku, Yokohama, 230-0045 Japan; 6 Division of Diabetes and Metabolism, The Institute for Adult Diseases, Asahi Life Foundation, 2-2-6 Nihonbashibakuro-cho, Chuo-ku, Tokyo, 103-0002 Japan; 7 AMED-CREST, Japan Agency for Medical Research and Development, 1-7-1 Otemachi, Chiyoda-ku, Tokyo, 100-0004 Japan; 8 Department of Prevention of Diabetes and Life-style Related Diseases, The University of Tokyo, 7-3-1 Hongo, Bunkyo-ku, Tokyo, 113-0033 Japan; 9 Department of Metabolism and Nutrition, Mizonokuchi Hospital, Faculty of Medicine, Teikyo University, 5-1-1 Futago, Takatsu-ku, Kawasaki, Kanagawa 213-8507 Japan; 10 Toranomon hospital, 2-2-2 Toranomon, Minato-ku, Tokyo, 105-8470 Japan

**Keywords:** Endocrinology, Type 2 diabetes, Drug development

## Abstract

Adiponectin receptors, AdipoR1 and AdipoR2 exert anti-diabetic effects. Although muscle-specific disruption of AdipoR1 has been shown to result in decreased insulin sensitivity and decreased exercise endurance, it remains to be determined whether upregulation of AdipoR1 could reverse them in obese diabetic mice. Here, we show that muscle-specific expression of human AdipoR1 increased expression levels of genes involved in mitochondrial biogenesis and oxidative stress-detoxification to almost the same extents as treadmill exercise, and concomitantly increased insulin sensitivity and exercise endurance in obese diabetic mice. Moreover, we created AdipoR-humanized mice which express human AdipoR1 in muscle of AdipoR1·R2 double-knockout mice. Most importantly, the small-molecule AdipoR agonist AdipoRon could exert its beneficial effects in muscle via human AdipoR, and increased insulin sensitivity and exercise endurance in AdipoR-humanized mice. This study suggests that expression of human AdipoR1 in skeletal muscle could be exercise-mimetics, and that AdipoRon could exert its beneficial effects via human AdipoR1.

## Abstract (web summary)

Masato Iwabu and Miki Okada-Iwabu et al. investigate whether diabetic phenotypes associated with disruption of the adiponectin receptor (AdipoR1) can be reversed in diabetic mice by upregulation of the receptor. They show that overexpressing human AdipoR1 in the muscles of diabetic mice increased insulin sensitivity and exercise endurance, suggesting a possible route for future clinical therapies.

## Introduction

Lack of exercise^1^ associated with increasing societal automation, as well as changes in dietary habits, has led to a drastic increase in the number of overweight individuals worldwide^2,3^. Obesity is known to cause insulin resistance, which is associated with type 2 diabetes and cardiovascular disease^4–6^, where it has become clear that decreased plasma adiponectin levels in obesity are causally implicated in these obesity-linked diseases^7–9^.

Adiponectin^10–13^ is a secreted protein highly expressed specifically in adipose tissue known as an adipokine^14–16^. Adiponectin, a member of the C1q/TNF related protein (CTRP) family that includes at least 15 other members (CTRP1-15) including some shown to regulate metabolism, has attracted much interest because of its anti-inflammatory and insulin-sensitizing effects^17^. Plasma levels of adiponectin have been shown to be decreased in obesity, insulin resistance, and type 2 diabetes, while adiponectin replenishment has been shown to improve insulin resistance and impaired glucose tolerance in mice^18–21^. Recent studies have suggested that CTRPs may also form heteromeric complexes, and that adiponectin can form heteromeric proteins with CTRP2 and CTRP9^17,22,23^.

We previously reported cloning of AdipoR1 and AdipoR2 as receptors for adiponectin^24^. AdipoR1 and AdipoR2 were assumed to have a seven-transmembrane (7TM) topology with an internal N-terminus and an external C-terminus, opposite to that of G-protein-coupled receptors (GPCRs)^24^. AdipoR1 and AdipoR2 serve as physiologically the most important receptors for adiponectin and play crucial roles in the regulation of glucose and lipid metabolism, as well as in inflammation and oxidative stress, in vivo^25^.

AdipoR1 may partially mediate the effects of CTRP9 on vascular endothelial cells and cardiomyocytes^22,26^. Since AdipoR1 and AdipoR2 belong to the 11-member PAQR family of transmembrane proteins^27^, it is speculated that other PAQR family members may constitute CTRP receptors^26^.

We have recently succeeded in determining the crystal structures of human AdipoR1 and AdipoR2, which revealed their novel structural and functional properties to the best of our knowledge, including their 7TM architecture and zinc-binding site, which were found to be clearly distinct from those of the GPCRs^28^. Of note, Vailiaukaité-Brooks et al. recently provided insight into the AdipoR1 and AdipoR2 structures and reported that AdipoRs exhibit ceramidase activity, while extremely low, concluding that further studies are required to fully characterize the enzymatic parameters and substrate specificity of AdipoRs^29^. In this regard, adiponectin administration has previously been reported to decrease ceramide levels in the liver and other tissues, and AdipoR-mediated ceramidase activity has been suggested to increase insulin sensitivity^30^. However, given that their catalytic activity is relatively low compared to other enzymes even after stimulation with adiponectin^29,31^. Therefore, it is suggested that AdipoR1 and/or AdipoR2 act on ceramides as any hydrolase and actually possess another lipid hydrolytic activity^31^. In addition, Scherer et al. described the possibility that AdipoRs possess another lipid hydrolase activity that could govern downstream signal transduction^32^. Thus, additional studies are required to define the enzymatic characteristics of these therapeutically important AdipoRs^31^. Moreover, the crystal structure of human alkaline ceramidase type 3 (ACER3) reported in 2018 was shown to possess seven alpha-helices forming a 7-transmembranes architecture with opposite N- and C-terminus domains, as with like AdipoRs^33^. While, the overall architecture of ACER3 was shown to be similar to the AdipoRs, however, significant/major differences were observed between ACER3 and AdipoRs^33^. These differences may have some impact on their intrinsic enzymatic properties of ACER3 versus AdipoRs, i.e. K_M_ parameters and substrate preference^33^.

AdipoR1 and AdipoR2 serve as major receptors for adiponectin in vivo, where AdipoR1 and AdipoR2 are shown to activate the AMPK^34^ and PPARα^35^ pathways, respectively^24,36,37^. AdipoR expression is shown to be decreased in obesity^38^, where analysis of systemic glucose and lipid metabolism in systemic AdipoR1·R2 double-knockout (DKO) mice demonstrated that the AdipoRs play a critical role in glucose and lipid metabolism^25,39^.

Then, analysis of muscle-specific AdipoR1-knockout mice showed that AdipoR1 in muscle is implicated in the regulation of mitochondrial function via Ca^2+^ signaling and the AMPK/SIRT1 pathways as well as insulin sensitivity and exercise endurance^40^. However, it remains to be determined whether upregulation of AdipoR1 in muscle to a physiological level is sufficient to ameliorate insulin resistance and impaired exercise endurance in obese diabetic mice.

Identifying ways to activate adiponectin/AdipoR signaling is expected to pave the way for the development of definitive therapies for the metabolic syndrome, type 2 diabetes, and atherosclerosis. In this connection, we have succeeded in identifying an orally active synthetic small-molecule AdipoR agonist, AdipoRon by screening a library of candidate compounds^39^. AdipoRon has been shown to exert anti-diabetic effects via mouse AdipoR, since AdipoRon failed to show any effect in DKO mice. Moreover, AdipoRon could prolong shortened lifespan observed in obese diabetic mice^39,41,42^. It is of extreme importance to clarify if AdipoRon may represent an anti-diabetic agent in humans as a step toward developing therapeutics for lifestyle-related diseases associated with obesity. However, it still remains unclear whether AdipoRon could actually activate the human AdipoRs, and at the same time exert anti-diabetic effects in vivo.

In this study, by genetically engineering muscle-specific human AdipoR1-transgenic (Tg) mice, we investigated whether augmentation of AdipoR1 expression in the muscle may have a similar role to exercise in muscle mitochondria synthesis and whether AdipoR1 signaling may be associated with anti-diabetic effects. We further engineered AdipoR1-humanized mice by cross-breeding muscle-specific human AdipoR1·Tg mice and DKO mice and investigated whether or not the small-molecule AdipoR agonist may act on human AdipoR1 thus exerting anti-diabetic effects.

## Results

### Increased muscle insulin sensitivity and exercise endurance in muscle-human AdipoR1·Tg mice

**Fig. 1:**
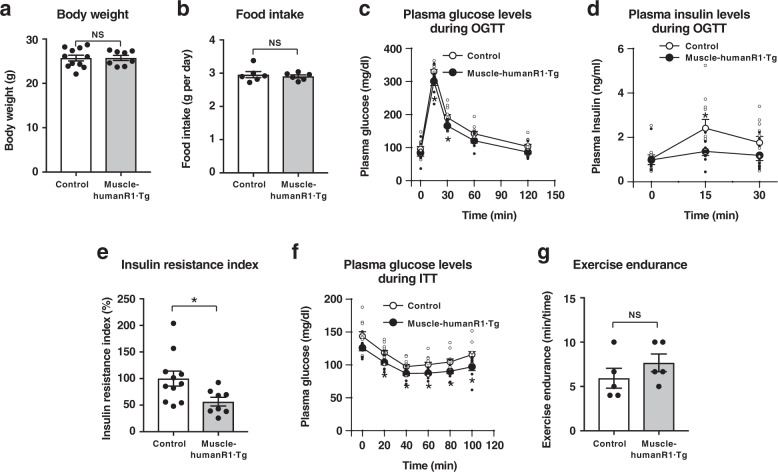
Muscle-specific upregulation of human AdipoR1 increases insulin sensitivity on a normal chow diet. Body weight (**a**), food intake (**b**), plasma glucose (**c**), plasma insulin (**d**) and insulin resistance index (**e**) during oral glucose tolerance test (OGTT) (1.5 g glucose per kg body weight), plasma glucose (**f**) during insulin tolerance test (ITT) (0.25 U insulin per kg body weight) and exercise endurance (**g**). All values are presented as means ± s.e.m. *P < 0.05 and **P < 0.01 compared to control mice (unpaired two-tailed t-test). NS, not significant. Control mice: n = 11; muscle-humanR1·Tg mice: *n* = 8 (**a**, **c**, **d**, **e**, **f**) *n* = 6 each (**b**), *n* = 5 each (**g**).

**Fig. 2:**
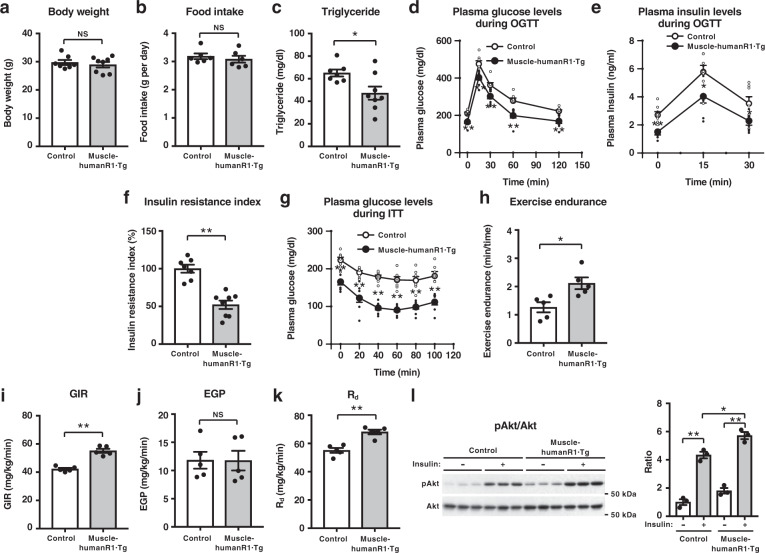
Muscle-specific upregulation of human AdipoR1 increases insulin sensitivity and exercise endurance on a high-fat diet. Body weight (**a**), food intake (**b**), plasma triglyceride (**c**), plasma glucose (**d**), plasma insulin (**e**), and insulin resistance index (**f**) during oral glucose tolerance test (OGTT) (1.0 g glucose per kg body weight), plasma glucose (**g**) during insulin tolerance test (ITT) (0.5 U insulin per kg body weight), exercise endurance (**h**), glucose infusion rate (GIR) (**i**), endogenous glucose production (EGP) (**j**) and rates of glucose disposal (R_d_) (**k**) during hyperinsulinemic euglycemic clamp study in control and muscle-humanR1·Tg mice on a high-fat diet. Phosphorylation and amount of Akt (**l**) in skeletal muscle treated with or without insulin (0.3 U per kg body weight) for 7.5 min in control and muscle-humanR1·Tg mice on a high-fat diet. All values are presented as means ± s.e.m. *P < 0.05 and **P < 0.01 compared to control mice (unpaired two-tailed t-test). NS, not significant. Control mice: *n* = 7; muscle-humanR1·Tg mice: *n* = 8 (**a**, **c**, **d**, **e**, **f**, **g**) *n* = 6 each (**b**), *n* = 5 each (**h**, **i**, **j**, **k**), *n* = 3 each (**l**).

In order to perform gain-of-function experiments to elucidate the physiological and pathophysiological roles of increased expression of human AdipoR1 in skeletal muscle in the regulation of metabolism on a long-term basis in vivo, we generated muscle-specific human AdipoR1·Tg (muscle-human R1·Tg) mice (Supplementary Fig. 1a). Several transgenic lines on a C57BL6 background (B6) were obtained. We quantitatively measured human ADIPOR1 and mouse AdipoR1 gene expression in skeletal muscle of muscle-human R1·Tg mice by real-time PCR analysis using a plasmid containing human and/or mouse AdipoR1 as a control. Real-time PCR analysis identified human ADIPOR1 mRNA to the ratio of approximately 0.7–1.3 folds (line 111 and line 241, respectively) of endogenous mouse mRNA in skeletal muscle (Supplementary Fig. 1b). Moreover, we determined the protein levels of human and mouse AdipoR1·R2 by western blots (Supplementary Fig. 1c, d), and found that the trends in protein levels of AdipoR1 were consistent with those in mRNA levels (Supplementary Fig. 1b). Furthermore, by western blots we confirmed abrogation of AdipoR1 and AdipoR2 proteins in AdipoR1·R2 DKO and expression of AdipoR1 protein, but not that of AdipoR2 protein, in AdipoR1-humanized mice (Supplementary Fig. 1d). We confirmed that both mice lines showed similar phenotypes in glucose tolerance and insulin resistance on both on the normal chow and high-fat diet (Figs. 1, 2, Supplementary Figs. 2, 3). Therefore, we carried out further experiments using line 241. Expression of human AdipoR1 within the physiological range in skeletal muscle did not significantly affect body weight (Fig. 1a) nor food intake (Fig. 1b) in mice on a normal chow diet, but it did significantly reduce plasma glucose levels at 15 and 30 min, tended to reduce plasma glucose levels at 60 and 120 min, and also significantly reduced plasma insulin levels at 15 min after oral glucose administration (Fig. 1c, d). Muscle-human R1·Tg mice fed a normal chow had a significantly reduced insulin resistance index (Fig. 1e). Moreover, muscle-human R1·Tg mice fed a normal chow diet tended to exhibit lower plasma glucose levels, and exhibited significantly lower plasma glucose levels than control mice during insulin tolerance tests (Fig. 1f). Furthermore, muscle-human R1·Tg mice fed a normal chow diet tended to exhibit increased exercise endurance (Fig. 1g).

We next tried to clarify the beneficial effects of upregulation of AdipoR1 in skeletal muscle in high-fat-diet-induced obese mice. Expression of human AdipoR1 in skeletal muscle did not significantly affect body weight (Fig. 2a) nor food intake (Fig. 2b) in mice on a high-fat diet, but it did significantly reduce fasting plasma triglyceride levels (Fig. 2c), plasma glucose and insulin levels before and during oral glucose tolerance tests (OGTTs) (Fig. 2d, e). The decrease in glucose levels in the face of reduced plasma insulin levels indicates improved insulin sensitivity, and the expression of human AdipoR1 in skeletal muscle indeed reduced insulin resistance index in mice on a high-fat diet (Fig. 2f). The glucose-lowering effect of exogenous insulin was also greater in muscle-human R1·Tg mice than in control mice fed a high-fat diet (Fig. 2g).

To study the impact of human AdipoR1 expression on skeletal muscle function in intact animals, we challenged control and muscle-human R1·Tg mice with involuntary physical exercise assessed by treadmill running. Importantly, muscle-human R1·Tg mice fed a high-fat diet exhibited significantly increased exercise endurance (Fig. 2h).

We next performed hyperinsulinaemic euglycaemic clamp experiment in mice on a high-fat diet. The glucose infusion rate was significantly increased (Fig. 2i), the endogenous glucose production was not altered (Fig. 2j), and the glucose disposal rate was significantly increased (Fig. 2k) in muscle-human R1·Tg mice fed a high-fat diet, indicating increased insulin sensitivity in muscle.

In agreement with the data obtained from the hyperinsulinaemic euglycaemic clamps, insulin-stimulated phosphorylation of Ser 473 in Akt was increased in skeletal muscle of muscle-human R1·Tg mice fed a high-fat diet (Fig. 2l).

### Increased PGC-1 and mitochondrial genes in muscle-human AdipoR1·Tg

**Fig. 3:**
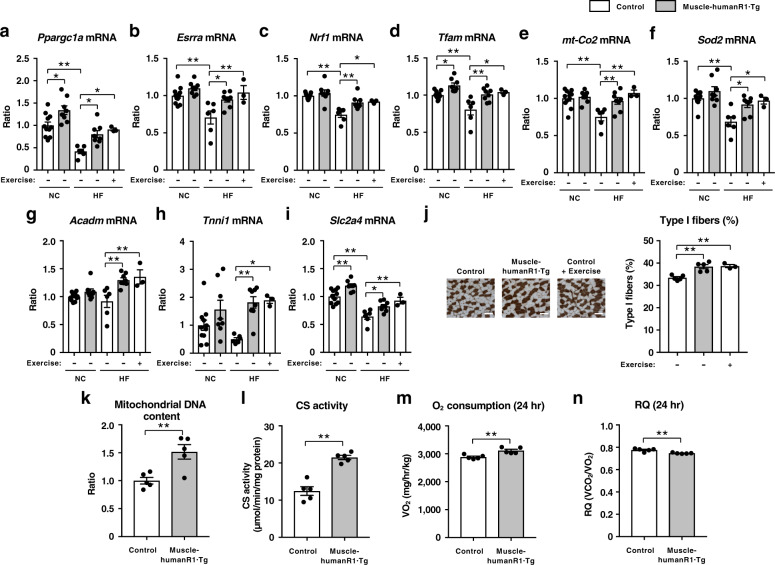
Muscle-specific upregulation of human AdipoR1 increases genes involved in mitochondrial biogenesis and oxidative stress-detoxification on a normal chow or a high-fat diet. *Ppargc1a* (**a**), *Esrra* (**b**), *Nrf1* (**c**), *Tfam* (**d**), *mt-Co2* (**e**), *Sod2* (**f**), *Acadm* (**g**), *Tnni1* (**h**) and *Slc2a4* (**i**) mRNA levels in skeletal muscle from control and muscle-humanR1·Tg mice on a normal chow (NC) or a high-fat (HF) diet. Control mice on a NC diet (*n* = 11); muscle-humanR1·Tg mice on a NC diet (*n* = 8). Control mice on a HF diet (*n* = 6); muscle-humanR1·Tg mice on a HF diet (*n* = 8); control mice on a HF diet after 2 weeks exercise (*n* = 3). ATPase (pH 4.3 for type I fibers) staining of soleus muscle (**j**) (scale bars, 100 µM), quantification of type I fibers based on fiber-type analyses in soleus muscle from control and muscle-humanR1·Tg mice on a HF diet and control mice on a HF diet after 2 weeks exercise. Control mice on a HF diet (*n* = 5); muscle-humanR1·Tg mice on a HF diet (n = 5); control mice on a HF diet after 2 weeks exercise (n = 3). Mitochondrial DNA content (**k**) as assessed by mitochondrial DNA copy number and citrate synthase (CS) enzyme activity (**l**) in skeletal muscle from control and muscle-humanR1·Tg mice on a HF diet. Control mice on a HF diet (*n* = 5); muscle-humanR1·Tg mice on a HF diet (*n* = 5). Oxygen (O_2_) consumption (**m**) and respiratory quotient (RQ) (**n**) in control and muscle-humanR1·Tg mice on a HF diet. *n* = 5 each. All values are presented as means ± s.e.m. *P < 0.05 and **P < 0.01 compared to control mice or as indicated. *P* values were determined ANOVA followed by Tukey–Kramer multiple comparison tests (**a**–**j)** or unpaired two-tailed t-test (**k**–**n**). NS, not significant.

To clarify the molecular mechanisms by which muscle-specific upregulation of AdipoR1 increases insulin sensitivity and exercise endurance, we performed gene expression analyses. High-fat diet significantly decreased the expression of genes involved in mitochondrial biogenesis such as *Ppargc1a*^43^(Fig. 3a) and estrogen-related receptor α (*Esrra*)^44^ (Fig. 3b), transcription such as nuclear respiratory factor 1 (*Nrf1*) (Fig. 3c), mitochondrial DNA replication/translation such as mitochondrial transcription factor A (*Tfam*) (Fig. 3d), oxidative phosphorylation such as cytochrome c oxidase subunit II (*mt-Co2*) (Fig. 3e), and mitochondrial oxidative stress-detoxification such as manganese superoxide dismutase (*Sod2*) (Fig. 3f).

Importantly, expression of human AdipoR1 in skeletal muscle of mice fed a high-fat diet significantly increased the expression of genes involved in mitochondrial biogenesis such as *Ppargc1a* (Fig. 3a) and *Esrra* (Fig. 3b), transcription such as *Nrf1* (Fig. 3c), mitochondrial DNA replication/translation such as *Tfam* (Fig. 3d), oxidative phosphorylation such as *mt-Co2* (Fig. 3e), and mitochondrial oxidative stress-detoxification such as *Sod2* (Fig. 3f).

We next examined the expression of metabolic genes and found that genes involved in fatty-acid oxidation, such as medium-chain acyl-CoA dehydrogenase (*Acadm*)^45^, were significantly increased in muscle-human R1·Tg mice fed a high-fat diet (Fig. 3g), which is consistent with significantly decreased plasma triglyceride levels (Fig. 2c).

The expression of human AdipoR1 in skeletal muscle of mice fed a high-fat diet increased the levels of the oxidative, high endurance type I fiber^46^ marker troponin I (slow) (*Tnni1*) (Fig. 3h), which was consistent with increased exercise endurance in muscle-human R1·Tg mice fed a high-fat diet (Fig. 2h). High-fat diet significantly decreased expression of the insulin-sensitive glucose transporter 4 (*Slc2a4*), while expression of human AdipoR1 in skeletal muscle of mice fed a high-fat diet increased its expression (Fig. 3i).

We next attempted to examine whether exercise could reverse the alterations of genes expression observed in high-fat-diet-induced obese diabetic mice. We challenged mice with involuntary physical exercise by treadmill running. The treadmill exercise regimen was 15 m min^−1^ for 30 min. Two weeks’ exercise significantly increased genes involved in mitochondrial biogenesis such as *Ppargc1a* (Fig. 3a) and *Esrra* (Fig. 3b), transcription such as *Nrf1* (Fig. 3c), mitochondrial DNA replication/translation such as *Tfam* (Fig. 3d), oxidative phosphorylation such as *mt-Co2* (Fig. 3e), mitochondrial oxidative stress-detoxification such as *Sod2* (Fig. 3f) as well as oxidative, high endurance type I fiber marker *Tnni1* (Fig. 3h) and *Slc2a4* (Fig. 3i) to almost the same extents as the expression of human AdipoR1 in skeletal muscle. These data revealed that the expression of human AdipoR1 and exercise-induced alterations in the expression of these genes in skeletal muscle to very similar extents, and that upregulation of AdipoR1 in skeletal muscle could be exercise-mimetics. These findings were consistent with those of the histological analysis also performed (Fig. 3j). In soleus muscle of muscle-human R1·Tg mice fed a high-fat diet, type I fibers were increased by 15% (Fig. 3j). Two weeks’ exercise significantly increased type I fibers in the soleus muscle of mice fed a high-fat diet (Fig. 3j).

Moreover, muscle-human R1·Tg mice fed a high-fat diet had an increased mitochondrial DNA content (Fig. 3k) and citrate synthase activity (Fig. 3l) in skeletal muscle. Furthermore, analysis of the mitochondrial morphology by electron microscopy revealed that exercise training increases skeletal muscle mitochondrial volume density by enlarging existing mitochondria^47^. The mitochondrial of skeletal muscle in muscle-human R1·Tg mice fed a high-fat diet appeared to be slightly enlarged compared to that of control mice, similar to exercise (Supplementary Fig. 4). These data indicated that the expression of human AdipoR1 increased mitochondria biogenesis in skeletal muscle.

Muscle-human R1·Tg mice fed a high-fat diet had significantly increased O_2_ consumption (Fig. 3m) but decreased RQ (Fig. 3n). These data suggested that the expression of human AdipoR1 in skeletal muscle resulted in increases in fatty-acid oxidation.

### Beneficial effects of AdipoRon via human AdipoR1 in skeletal muscle

We previously showed that orally active AdipoR agonist AdipoRon ameliorated insulin resistance and glucose intolerance in mice fed a high-fat diet, which was completely obliterated in AdipoR1 and AdipoR2 DKO (mouse R1·R2DKO) mice^39^. For the development of clinically available drug, it is extremely important to clarify whether AdipoRon could exert its beneficial effects via human AdipoR. To address this critically important issue, we next generated mouse R1·R2DKO mice expressing human AdipoR1 in skeletal muscle (AdipoR1-humanized mice).

We next studied the dose-dependent effects of AdipoRon in AdipoR1-humanized mice fed a high-fat diet. Orally administered AdipoRon reduced plasma glucose levels in a dose-dependent manner (Supplementary Fig. 5a). Furthermore, when orally administered at higher doses, AdipoRon (100–250 mg per kg body weight) did not cause hypoglycaemia, and observed no abnormality and no signs of toxicity (Supplementary Fig. 5b).

**Fig. 4:**
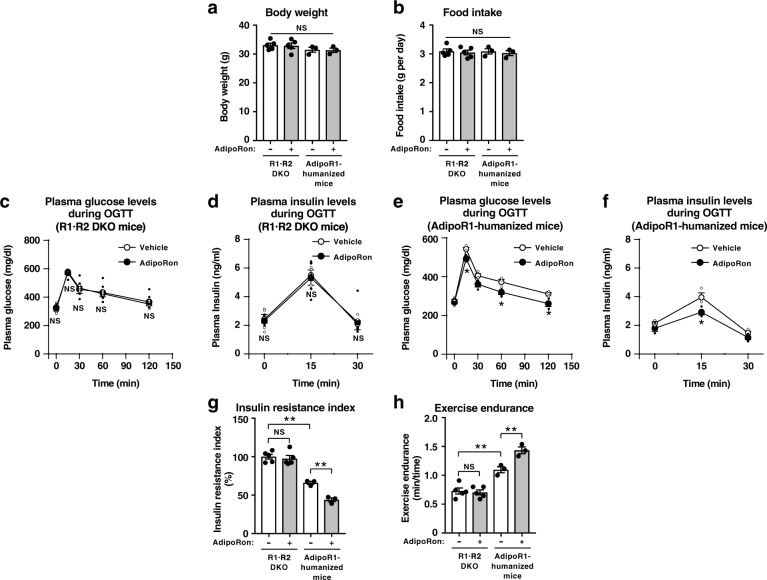
AdipoRon could increase insulin sensitivity and exercise endurance in AdipoR-humanized mice on a high-fat diet. Body weight (**a**), food intake (**b**), plasma glucose (**c**, **e**), plasma insulin (**d**, **f**) and insulin resistance index (**g**) during oral glucose tolerance test (OGTT) (1.0 g glucose per kg body weight), exercise endurance (**h**) in mouse R1·R2DKO mice and muscle-humanR1·Tg/mouse R1·R2DKO (AdipoR1-humanized) mice on a high-fat diet, treated once daily oral administration of AdipoRon (50 mg per kg body weight) for two weeks. All values are presented as means ± s.e.m. *P < 0.05 and **P < 0.01 compared to control mice or as indicated. *P* values were determined ANOVA followed by Tukey–Kramer multiple comparison tests (**a**, **b**, **g**, **h)** or unpaired two-tailed t-test (**c**–**f**). NS, not significant. R1·R2DKO mice (Vehicle: *n* = 5; AdipoRon: *n* = 5), muscle-AdipoR1-humanized mice (Vehicle: *n* = 3; AdipoRon: *n* = 3).

As we previously showed, oral administration of AdipoRon (50 mg per kg body weight) for 10 days had no effects on body weight (Fig. 4a) nor food intake (Fig. 4b), and could not ameliorate hyperglycemia (Fig. 4c) nor hyperinsulinemia (Fig. 4d) in mouse R1·R2DKO mice fed a high-fat diet. AdipoRon also had no effects on body weight (Fig. 4a) nor food intake (Fig. 4b) in AdipoR1-humanized mice fed a high-fat diet, importantly, AdipoRon could significantly decrease plasma glucose levels (Fig. 4e) and plasma insulin levels (Fig. 4f) during OGTT and also insulin resistance index (Fig. 4g), indicating that AdipoRon could indeed ameliorate insulin resistance and glucose intolerance via human AdipoR1 in skeletal muscle. The expression of human AdipoR1 in skeletal muscle in mouse R1·R2DKO mice ameliorated insulin resistance and increased exercise endurance (Fig. 4g, h).

We challenged mice fed a high-fat diet with involuntary physical exercise by treadmill running and then assessed muscle endurance. AdipoRon significantly increased exercise endurance in AdipoR1-humanized mice, but not in R1·R2DKO mice fed a high-fat diet (Fig. 4h). These data clearly indicated that AdipoRon could increase exercise endurance via human AdipoR1 in the skeletal muscle of mice fed a high-fat diet.

**Fig. 5:**
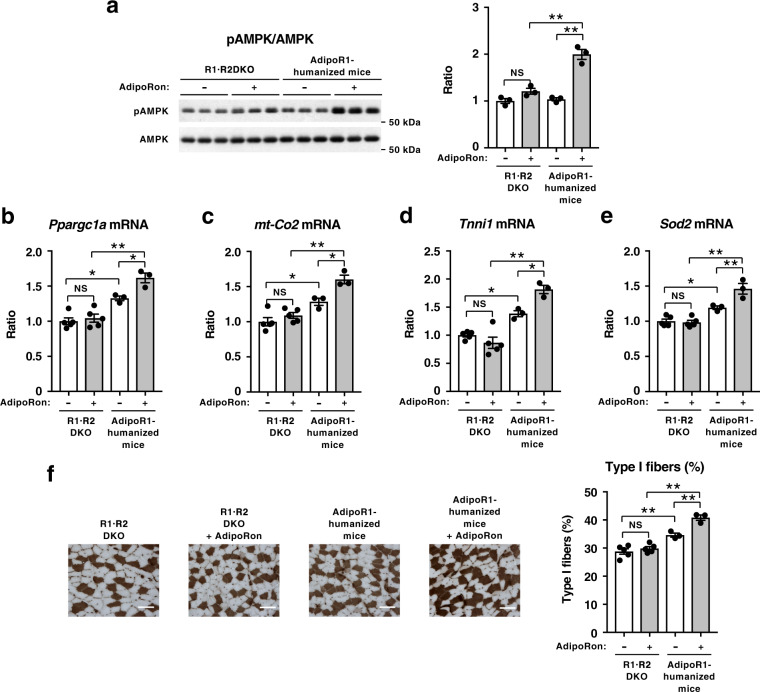
AdipoRon could increase pAMPK and genes involved in mitochondrial biogenesis as well as oxidative stress-detoxification in AdipoR-humanized mice on a high-fat diet. Phosphorylation and amount of AMPK (**a**) in skeletal muscle from R1·R2DKO mice or AdipoR1-humanized mice on a high-fat diet, treated for 7.5 min after injection of 50 mg of AdipoRon per kg body weight intravenously into mice through an inferior vena cava catheter. *Ppargc1a* (**b**), *mt-Co2* (**c**), *Tnni1* (**d**) and *Sod2* (**e**) mRNA levels in skeletal muscle from R1·R2DKO mice or AdipoR1-humanized mice on a high-fat diet, treated once daily oral administration of AdipoRon (50 mg per kg body weight) for two weeks. ATPase (pH 4.3 for type I fibers) staining of soleus muscle (**f**) (scale bars, 100 µM), quantification of type I fibers based on fiber-type analyses in soleus muscle from R1·R2DKO mice and AdipoR1-humanized mice on a high-fat diet with or without AdipoRon (50 mg per kg body weight). All values are presented as means ± s.e.m. *P < 0.05 and **P < 0.01 compared to control mice or as indicated (ANOVA followed by Tukey–Kramer multiple comparison tests). NS, not significant. *n* = 3 each (**a**) R1·R2DKO mice (Vehicle: *n* = 5; AdipoRon: *n* = 5), AdipoR1-humanized mice (Vehicle, *n* = 3; AdipoRon, *n* = 3) (**b**–**f**).

Intravenous injection of AdipoRon (50 mg per kg body weight) significantly induced phosphorylation of AMPK in skeletal muscle of AdipoR1-humanized mice, but not in R1·R2DKO mice (Fig. 5a) fed a high-fat diet. These data clearly indicated that AdipoRon could activate AMPK in skeletal muscle via human AdipoR1 in skeletal muscle of mice fed a high-fat diet.

Orally administered AdipoRon significantly increased expression levels of genes involved in mitochondrial biogenesis such as *Ppargc1a* (Fig. 5b) and genes involved in oxidative phosphorylation such as *mt-Co2* (Fig. 5c) in skeletal muscle of AdipoR1-humanized mice, but not in R1·R2DKO mice fed a high-fat diet. Moreover, AdipoRon significantly increased expression levels of oxidative, high endurance type I fiber marker *Tnni1* (Fig. 5d) and genes involved in mitochondrial oxidative stress-detoxification such as *Sod2* (Fig. 5e) in skeletal muscle of AdipoR1-humanized mice, but not in R1·R2DKO mice fed a high-fat diet. Treatment of R1·R2DKO mice with AdipoRon failed to increase type I fibers in skeletal muscle (Fig. 5f). AdipoRon significantly increased the ratio of type I fibers in skeletal muscle of AdipoR1-humanized mice (Fig. 5f). These data clearly indicated that AdipoRon could exert its beneficial effects in skeletal muscle via human AdipoR1 in skeletal muscle of mice fed a high-fat diet.

We found that AdipoRon significantly decreased the expression of *Pck1* and *G6pc* in the liver of wild-type mice but not in that of R1·R2DKO mice fed a high-fat diet^39^. AdipoRon increased the expression levels of the gene encoding PPARα itself and its target genes, including those involved in fatty-acid combustion such as acyl-CoA oxidase (*Acox1*), those involved in energy dissipation such as uncoupling protein 2 (*Ucp2*) in the liver of wild-type mice but not in that of R1·R2DKO mice fed a high-fat diet^39^. However, the expression of human AdipoR1 in skeletal muscle of R1·R2DKO mice caused no change in the expression of *Pck1*, *G6pc*, *Acox1*, and *Ucp2* in the liver (Supplementary Fig. 6a–d).

AdipoRon reduced the expression levels of genes encoding pro-inflammatory cytokines such as *Tnf* and *Ccl2* in the epididymal white adipose tissue (eWAT) of AdipoR1-humanized mice but not in that of R1·R2DKO mice fed a high-fat diet (Supplementary Fig. 6e, f). Moreover, AdipoRon reduced the levels of markers for classically activated M1 macrophages such as CD11c (*Itgax*), but not the levels of markers for the alternatively activated M2 macrophages such as CD206 (*Mrc1*) in the eWAT of AdipoR1-humanized mic fed a high-fat diet, while these changes were not observed in R1·R2DKO mice (Supplementary Fig. 6g, h). In contrast to eWAT, no appreciable change was seen in expression levels of genes encoding pro-inflammatory cytokines and macrophage markers in the inguinal white adipose tissue (iWAT) of AdipoR1-humanized mice (Supplementary Fig. 6i–l). These data suggested that the expression of human AdipoR1 in skeletal muscle resulted in increases anti-inflammatory effects in eWAT.

## Discussion

We engineered muscle-specific human AdipoR1-transgenic mice in this study and demonstrated that enhancing AdipoR1 expression in muscle play a role in the regulation of mitochondria-related genes and that it also improves impaired glucose tolerance and insulin resistance in mouse models of obesity and type 2 diabetes. PGC-1α serves as a key regulator of mitochondrial biogenesis and function in muscle^43,48^. Of note here is the fact that the expression of AdipoR1, as well as that of PGC-1α, has been reported to be decreased in obese, diabetic db/db mice or in type 2 diabetic patients^40,49^. Our present experiments also showed that the expression of AdipoR1, PGC-1α and mitochondria-related genes was decreased in mice fed a high-fat diet. Importantly, the co-expression of both human AdipoR1 and mouse endogenous AdipoR1, yielding a 2-fold upregulation of AdipoR1, was shown to result in significantly increased expression of PGC-1α and mitochondria-related genes, as well as increased molecules involved in mitochondrial oxidative stress-detoxification. These alterations collectively resulted in improvements in impaired glucose tolerance and insulin resistance in mice fed a high-fat diet. Thus, we have demonstrated for the first time in this study that the upregulation of AdipoR1 in muscle is sufficient to ameliorate insulin resistance and impaired exercise endurance in obese diabetic mice.

Exercise has been reported to activate the Ca^2+^ signaling, AMPK, and PGC-1α pathways, and at the same time improve glucose tolerance and insulin resistance. Thus exercise is likely to exert beneficial effects on obesity-related disease, such as type 2 diabetes, and may contribute to healthy longevity^1,40,48,50,51^. Strikingly, our study showed that muscle-specific AdipoR1 upregulation led to the expression of mitochondria-related genes being recovered, with the extent of their recovery found to be similar to that in high-fat diet-fed mice subjected to exercise, suggesting that upregulation of AdipoR1 may represent “exercise-mimetics”.

Incidentally, of note, another group has reported that their AdipoR1·R2 DKO mice died during their development^52^ and that they were derived from AdipoR2-knockout mice exhibiting a phonotype opposite to that of adiponectin-knockout mice characterized by aggravating of insulin resistance and atherosclerosis^53–55^. Thus, the difference between the two AdipoR1·R2 DKO mice seems to be due to differences in their knockout mice constructs. Indeed, we have demonstrated that neither AdipoR1 nor AdipoR2 is expressed in AdipoR1·R2 DKO mice (Supplementary Fig. 1d). On the other hand, Lindgren et al. used heterozygous AdipoR1 and AdipoR2 gene knockout mice obtained from Deltagen (San Carlos, CA) in their experiments^52^. Briefly, the mice were generated by gene targeting through homologous recombination in 129/OlaHsd ES cells using target vectors that contained the following cassette: lacO-splice acceptor–internal ribosomal entry site–lacZ–poly A–phosphor glycerate kinase promoter–neomycin resistance–poly A (PGK-neo)^52^. Very importantly, the other allele, which retained PGK-neo and led to additional phenotypes, demonstrating that the expression of other genes was affected^56–58^. It was thus suggested that the AdipoR1·R2 DKO mice used by Lindgren et al.^52^ may have affected the expression of other genes besides AdipoR1·R2.

The metabolic syndrome and type 2 diabetes are counted among the major causes of cardiovascular disease and cancer as leading causes of death in humans^59–61^, which has led to mounting expectations for radical therapeutics for obesity-related diseases, such as type 2 diabetes. Despite the wide range of therapeutics aimed at improving insulin resistance currently available, however, the number of type 2 diabetic patients continues to increase, suggesting that there still remains a considerable unmet need in anti-diabetic treatment. Against this background, small-molecule AdipoR agonists are drawing attention as a potential another class of drugs for obesity-related diseases including type 2 diabetes^62,63^. We have demonstrated for the first time in this study that one of these compounds, AdipoRon, acts on human AdipoR1, activates the AMPK pathway downstream, and augments the expression of mitochondria-related genes, thus improving impaired glucose tolerance and insulin resistance in vivo. These findings appear to provide proof of concept that the small-molecule AdipoR agonist may have the potential to work not only in mice but in humans, whose observation is required for its clinical development. By measuring molecular interactions using a Biacore system, AdipoRon has been shown to directly bind to human AdipoRs in vitro^39^.

After oral administration of 50 mg per kg body weight of AdipoRon to C57BL/6 wild-type mice, we found that the half-life of AdipRon was 2 h, and the maximal concentration (*C*_max_) of AdipoRon was 11.8 µM^39^. The half-life of the glucose-lowering effect of AdipoRon was 8 h. After the maximum concentration was reached at 30 min, the glucose-lowering effect became maximal at 2 h, and lasted for at least 8 h and even after AdipoRon was cleared from plasma. Importantly, AdipoRon did prolong the shortened lifespan of obese diabetic mice.

Based on the information obtained from structures of AdipoRs^28^, much progress has recently been made in the analysis of the co-crystal structure of AdipoRs and AdipoRon as well as in the development of small-molecule AdipoR agonists for clinical use. Moreover, expectations are mounting for the development of small-molecule AdipoR agonists for use as anti-diabetic agents.

Thus, the small-molecule AdipoR agonist appears to promise to become an effective therapeutic modality for lifestyle-related diseases that have obesity as a common underlying cause, promoting healthy longevity in humans.

## Methods

### Mouse studies

Male mice were 6–10 weeks of age at the time of the experiment. The animal care and use procedures were approved by the Animal Care Committee of the University of Tokyo. They were housed in cages and maintained on a 12 h light-dark cycle with access to chow and water ad libitum. In these experiments, we used standard chow diet with the following composition: 25.6% (wt/wt) protein, 3.8% fiber, 6.9% ash, 50.5% carbohydrates, 4% fat and 9.2% water (CE-2, CLEA Japan Inc.) or high-fat diet 32 consisting of 25.5% (wt/wt) protein, 2.9% fiber, 4.0% ash, 29.4% carbohydrates, 32% fat and 6.2% water (CLEA Japan Inc.)^64^. Plasma glucose levels were determined by using a glucose B-test (Wako Pure Chemical Industries). Plasma insulin was measured with an insulin immunoassay (Shibayagi). Plasma triglyceride (Wako Pure Chemical Industries) was assayed by enzymatic methods. All measurements were performed in blinded fashion. When the mice in the same genotype received different treatments, they were randomly assigned to treatment groups.

### Administration of AdipoRon

AdipoRon (Purity, 95%) was synthesized by Enamine Ltd (Kyiv, Ukraine)^39^. For administration, AdipoRon was prepared in 0.5% hydroxypropyl methylcellulose. AdipoRon (50 mg per kg body weight) was orally administered to mice once daily for two weeks. The sampling of the mice parameters was performed 4 h after oral administration. To study AMPK phosphorylation in vivo, AdipoRon was intravenously injected at 50 mg per kg body weight into the mice through an inferior vena cava catheter. The mice were treated for 7.5 min with AdipoRon and were examined phosphorylation and amount of AMPK in skeletal muscle.

### Generation of AdipoR1-humanized mice

A human AdipoR1 coding sequence cDNA^24^ for insertion was cloned and the transgene vector CAG-LacZ-human AdipoR1, which contains a CAG gene promotor-loxP-LacZ gene-loxP-human AdipoR1 was constructed (Supplementary Fig. 1). The *ADIPOR1* transgene was microinjected into a fertilized mouse egg to produce a transgenic mouse line. Animals transgenically expressing cre recombinase under the control of the muscle creatine kinase promoter (MCK-Cre) were generous gifts from Dr. C. R. Kahn of the Joslin Diabetes Center, Boston, MA, USA^40^. We backcrossed them with C57Bl/6 mice more than seven times. CAG-LacZ-human AdipoR1 mice were mated with MCK-Cre mice to generated CAG-LacZ-human AdipoR1/MCK-Cre double transgenic mice. The original *Adipor1*^+/−^/*Adipor2*^+/−^ mice (C57BL/6 and 129/Sv mixed background)^25^ were backcrossed with C57Bl/6 mice more than seven times. *Adipor1*^−/−^/*Adipor2*^−/−^ mice were prepared by *Adipor1*^+/−^/*Adipor2*^+/−^ mouse intercrosses^25^. CAG-LacZ-human AdipoR1/MCK-Cre/AdipoR1^−/−^AdipoR2^−/−^ DKO mice (AdipoR1-humanized mice) were generated by crossing CAG-LacZ-human AdipoR1/MCK-Cre double transgenic mice to *Adipor1*^−/−^/*Adipor2*^−/−^ mice. Genotyping of the mice was performed using PCR. Three primers were used for *Adipor1* genotyping: the forward primer 1 (5′-GCAGGGTAAGCTGATTAGCTATG-3′), the forward primer 2 (5′-ATAGATCTCTCGTGGGATCATTG-3′), and the reverse primer (5′-TTACTGCACTTCTTCTGCTGGA-3′). Three primers were used for Adipor2 genotyping: the forward primer 1 (5′-AGCCTACTGCCTACTGTATTGT-3′), the forward primer 2 (5′-ATAGATCTCTCGTGGGATCATTG-3′), and the reverse primer (5′-ACTCTTCTAACCTTCATCAGGAG-3′). The primers for detecting rabbit beta-globin polyA were the forward primer (5′-CTGCTGTCCATTCCTTATTCC-3′) and the reverse primer (5′-GGACAGCTATGACTGGGAGTAG-3′). The primers were used for detection of Cre were 5′-CGCCGCATAACCAGTGAAAC-3′, 5′-ATGTCCAATTTACTGACCG-3′.

### Insulin resistance index

The OGTT was conducted as previously described^18,36^ with slight modifications. The areas of the glucose and insulin curves were calculated by multiplying the cumulative mean height of the glucose values (1 mg ml^−1^ = 1 cm) and insulin values (1 ng ml^−1^ = 1 cm), respectively, by time (60 min = 1 cm)^18,25^. The insulin resistance index was calculated from the product of the areas of glucose and insulin × 10^−2^ in the glucose tolerance test. The results are expressed as the percentage of the value of control mice^18,25^.

### Hyperinsulinemic euglycemic clamp study

Clamp studies^39,40,64,65^ were carried out in mice on a high-fat diet. An infusion catheter was inserted into the right jugular vein under general anesthesia with sodium pentobarbital, 2–3 days before the study. Studies were performed on mice under conscious and unstressed conditions after a 6 h fast. A primed continuous infusion of insulin (Humulin R, Lilly) was given (10.0 milliunits kg^−1^ min^−1^), and the blood glucose concentration, monitored every 5 min, was maintained at 120 mg per dl by the administration of glucose (5 g of glucose per 10 ml enriched to 20% with [6,6-^2^H_2_] glucose (Sigma)) for 120 min. Blood was sampled via tail tip bleeds at 90, 105, and 120 min for determination of the rate of glucose disappearance (Rd). Rd was calculated according to nonsteady-state equations, and endogenous glucose production (EGP) was calculated as the difference between Rd and exogenous glucose infusion rates^39,40,64,65^.

### Measurement of exercise capacity in mice

The treadmill exercise regimen was 15 m min^−1^ for 30 min per day for 2 weeks. Exercise endurance was assessed by dividing 20 min by the number of times a mouse was unable to avoid electrical shocks.

### Real-time PCR

Real-time PCR was performed according to the method described previously^25,40^. Total RNA was prepared from cells with ISOGEN (Nippon Gene), according to the manufacturer’s instructions. We used the real-time PCR method to quantify the mRNAs^24^, with slight modifications. The real-time PCR was performed using specific TaqMan Gene Expression Assays (Applied Biosystems) for *Adipor1* (Mm01291334_mH), *Ppargc1a* (Mm00447183_m1), *Esrra* (Mm00433143_m1), *Nrf1* (Mm00447996_m1), *Tfam* (Mm00447485_m1), *mt-Co2*(Mm03294838_g1), *Sod2* (Mm00449726_m1), *Acadm* (Mm00431611_m1), *Tnni1* (Mm00502426_m1), *Slc2a4* (Mm00436615_m1), *Pck1* (Mm00440636_m1), *G6pc* (Mm00839363_m1), *Acox1* (Mm00443579_m1)*, Ucp2* (Mm00495907_g1)*, Tnf* (Mm00443258_m1)*, Ccl2* (Mm00441242_m1)*, Itgax* (Mm01271275_m1)*, Mrc1* (Mm01329361_m1) and ADIPOR1 (forward-Primer: 5′-TCCTAAGCACCGGCAGACA-3′, reverse-Primer: 5′-GGCACGACGCCACTCAAG -3′, Probe: 5′-AGCAGGCGTGTTCC-3′)

### Western blot analysis

To determine insulin-stimulated phosphorylation of Ser 473 in Akt in skeletal muscle, we injected 0.3 U of insulin per kg body weight intravenously into mice through an inferior vena cava catheter. A western blot analyses were performed with anti-phosphorylated-Akt (Ser 473) (Cell Signaling Technology, 1:1000; #4060) and anti-Akt (Cell Signaling Technology, 1:1000; #9272) antibodies. To study AMPK phosphorylation in vivo, we injected 50 mg of AdipoRon per kg body weight intravenously into mice through an inferior vena cava catheter^36,39^. Phosphorylation and protein levels of αAMPK^66–69^ were determined. Western blot analyses were performed with anti-phosphorylated-AMPK (Cell Signaling Technology #2535) and anti-αAMPK (Cell Signaling Technology, 1:1000; #2532) antibodies. To determine protein levels of AdipoR1 and AdipoR2, western blot analyses were performed with anti-AdipoR1 antibody (IBL, 1:1000; #18993), anti-AdipoR2 antibody (IBL, 1:1000; #18995), and anti-alpha-Tubulin (Cell Signaling Technology, 1:1000; #2125). Uncropped images of western blotting are shown in Supplementary Fig. 7.

### Mitochondrial content assay

Mitochondrial content assays^40,70^ were carried out. For quantification of mitochondrial content, mtDNA in skeletal muscle was determined by qPCR^71^.

### Citrate synthase activity

Citrate synthase activity was determined spectrophotometrically from the homogenates by using previously described methods^47,70,72^. Briefly, measurements of citrate synthase activity were carried out at room temperature in 0.1 ml of assay medium containing 10 µg of muscle homogenate in the presence of saturating concentrations of 0.3 mM acetyl-coenzyme A, 0.1 mM dithionitrobenzoic acid and 0.5 mM oxaloacetate. The reaction was initiated by adding 0.5 mM oxaloacetate and monitored by measuring the increase in absorbance at 412 nm due to thionitrobenzoic acid formation. All activities were normalized to mg of total protein.

### Histological analyses

Histological analyses were performed according to the method described previously^40^. We examined the relationship between the change in type I fiber gene expression and actual muscle fiber morphology in the skeletal muscle (soleus) of muscle-humanR1·Tg mice by using light microscopy and histochemical analysis. Samples of the muscle were frozen in liquid nitrogen-cooled isopentane. Type I and type II fibers were differentiated with myosin ATPase staining at different pH values. Specifically, at pH 4.3, type I fibers were well stained while type II fibers were not^46^.

### Oxygen consumption

Oxygen consumption was measured with an O_2_/CO_2_ metabolism measurement system for 24 h in the animals under ad libitum feeding conditions (Model MK-5000RQ; Muromachikikai, Tokyo, Japan). Each mouse was placed in a sealed chamber (560 ml volume) with an air flow of 0.65 l min^−1^ for 24 h at room temperature. The oxygen consumed was converted to milliliters per minute by multiplying it by the flow rate.

### Analyses of mitochondrial morphology by the electron microscopy

The samples were fixed with 2% paraformaldehyde and 2% glutaraldehyde in 0.1 M phosphate buffer (PB), pH 7.4 at 4 °C overnight. After this fixation the samples were washed 3 times with 0.1 M PB for 30 min each, and were postfixed with 2 % osmium tetroxide in 0.1 M PB at 4 °C for 2 h. The samples were then dehydrated in graded ethanol solutions (50, 70, 90, 100%). The schedule was as follows: 50% and 70% for 30 min each at 4 °C, 90% for 30 min at room temperature, and 4 changes of 100% for 30 min each at room temperature. The samples were infiltrated with propylene oxide (PO) 2 times for 30 min each and were put into a 70:30 mixture of PO and resin (Quetol-812; Nisshin EM Co., Tokyo, Japan) for 1 h, then with the cap tubes left open, PO was volatilized overnight. The samples were transferred to a fresh 100% resin, and were polymerized at 60 °C for 48 h. The polymerized resins were ultra-thin sectioned at 70 nm with a diamond knife using an ultramicrotome (Ultracut UCT; Leica, Vienna, Austria) and the sections were mounted on copper grids. They were stained with 2% uranyl acetate at room temperature for 15 min, and then were washed with distilled water and secondary-stained with Lead stain solution (Sigma-Aldrich Co., Tokyo, Japan) at room temperature for 3 min. The grids were observed by a transmission electron microscope (JEM-1400Plus; JEOL Ltd., Tokyo, Japan) at an acceleration voltage of 100 kV. Digital images (3296 × 2472 pixels) were taken with a CCD camera (EM-14830RUBY2; JEOL Ltd., Tokyo, Japan).

### Statistics and reproducibility

Results are expressed as mean as ± s.e.m. Differences between two groups were assessed using unpaired two-tailed t-tests. Data involving more than two groups were assessed by analysis of variance (ANOVA) followed by Tukey-Kramer multiple comparison tests. Representative data from one of 2–5 independent experiments are shown. Every experiment was performed multiple times with essentially the same results.

### Reporting summary

Further information on research design is available in the Nature Research Reporting Summary linked to this article.

## Supplementary information

Supplementary Information

Description of Additional Supplementary Files

Supplematary Data 1

Reporting Summary

## Data Availability

The source data for the figures are available in Supplementary Data 1. Full blots are shown in Supplementary Information. All other data that support the findings of this study are available from the corresponding author on reasonable request.
