# Supplementary material for: AdipoR agonist increases insulin sensitivity and exercise endurance in AdipoR-humanized mice
Source: Commun Biol. 2021 Jan 8;4:45. doi: 10.1038/s42003-020-01579-9 (PMC7794315; doi:10.1038/s42003-020-01579-9)
Supplement: Supplementary file 1 — Supplementary Information [file 42003_2020_1579_MOESM1_ESM.pdf]

Supplementary Information

AdipoR agonist increases insulin sensitivity and exercise endurance in AdipoR-humanized mice

Masato Iwabu[1,2,3+], **Miki Okada-Iwabu**[1,3+*], **Hiroaki Tanabe**[4,5], **Nozomi Ohuchi**[1], **Keiko Miyata**[1], **Toshiko Kobori**[6], **Sara Odawara**[1], **Yuri Kadowaki**[1], **Shigeyuki Yokoyama**[4,5], **Toshimasa Yamauchi**[1,7*], **and Takashi Kadowaki**[1,8,9,10*]

[1] Department of Diabetes and Metabolic Diseases, Graduate School of Medicine, The University of Tokyo, 7-3-1 Hongo, Bunkyo-ku, Tokyo 113-0033, Japan.
[2] PRESTO, Japan Science and Technology Agency, 4-1-8 Honcho, Kawaguchi, Saitama 332-0012, Japan.
[3] Laboratory for Advanced Research on Pathophysiology of Metabolic Diseases, The University of Tokyo, 7-3-1 Hongo, Bunkyo-ku, Tokyo 113-0033, Japan.
[4] RIKEN Structural Biology Laboratory, 1-7-22 Suehiro-cho, Tsurumi-ku, Yokohama 230-0045, Japan.
[5] RIKEN Cluster for Science, Technology and Innovation Hub, 1-7-22 Suehiro-cho, Tsurumi-ku, Yokohama 230-0045, Japan.
[6] Division of Diabetes and Metabolism, The Institute for Adult Diseases, Asahi Life Foundation, 2-2-6 Nihonbashibakuro-cho, Chuo-ku, Tokyo 103-0002, Japan.
[7] AMED-CREST, Japan Agency for Medical Research and Development, 1-7-1 Otemachi, Chiyoda-ku, Tokyo 100-0004, Japan.
[8] Department of Prevention of Diabetes and Life-style Related Diseases, The University of Tokyo, 7-3-1 Hongo, Bunkyo-ku, Tokyo 113-0033, Japan.
[9] Department of Metabolism and Nutrition, Mizonokuchi Hospital, Faculty of Medicine, Teikyo University, 5-1-1 Futago, Takatsu-ku, Kawasaki, Kanagawa 213-8507, Japan.
[10] Toranomon hospital, 2-2-2 Toranomon, Minato-ku, Tokyo 105-8470, Japan.

[*]Correspondence and requests for materials should be addressed to T.Kadowaki (email: t-kadowaki@toranomon.kkr.or.jp) or T.Y. (email: tyamau-tky@umin.ac.jp) or M.O.-I. (email: omiki-tky@ umin.ac.jp)
[+]These authors contributed equally to this work.

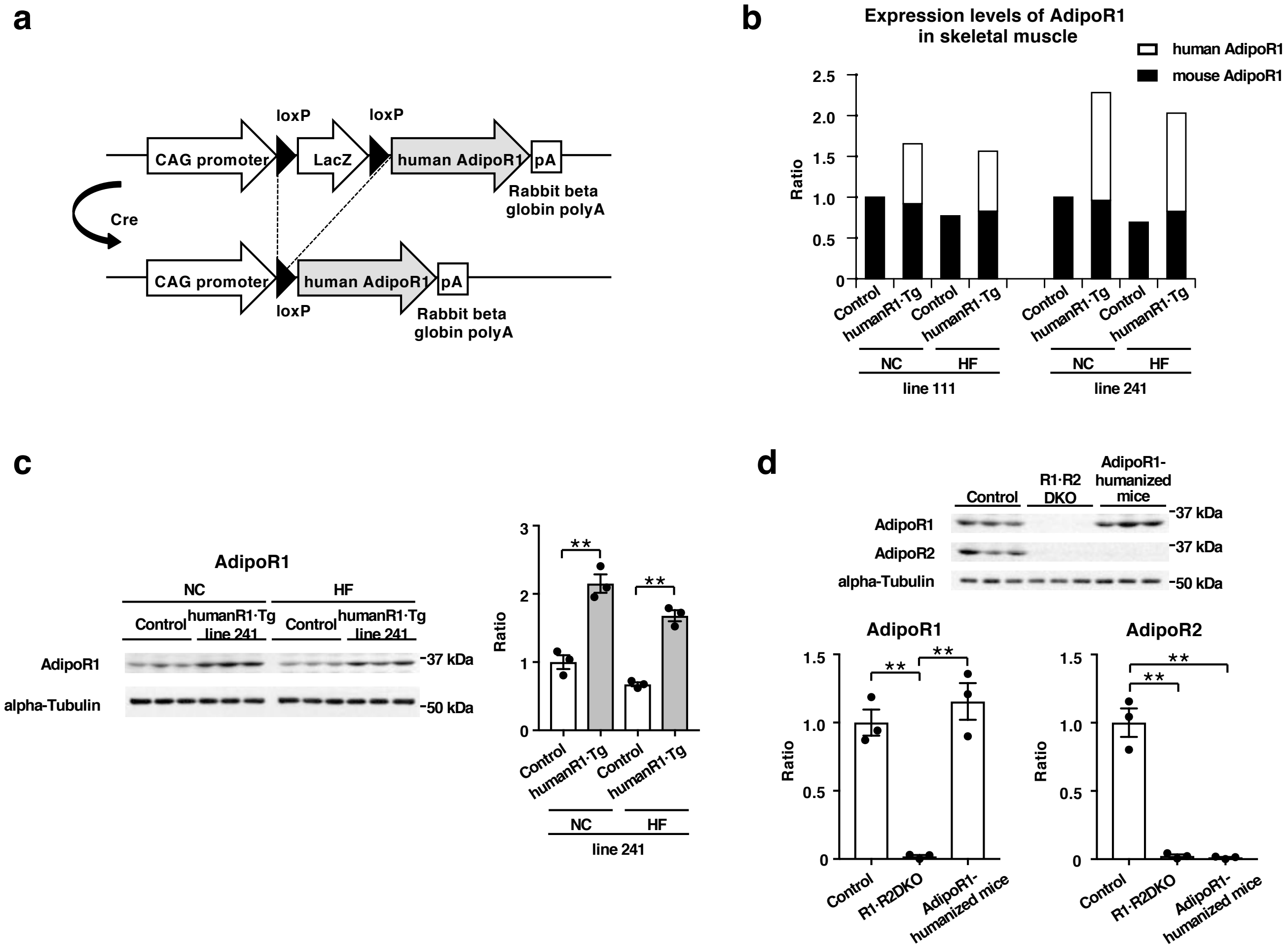

Supplementary Figure 1 | Generation of two independent mouse lines increased human AdipoR1 in skeletal muscle.
(**a**) Plasmid construction and generation of transgenic mice. The conditional transgenic human AdipoR1 transgene consists of a CAG promoter, a loxP-flanked LacZ gene, followed by human AdipoR1 cDNA. MCK-Cre-mediated recombination excises the LacZ gene, resulting in the expression of human AdopoR1. (**b**) Expression levels of human and mouse AdipoR1 in skeletal muscle. Human AdipoR1 (open bars) and mouse AdipoR1 (solid bars) mRNA levels in skeletal muscle from muscle-humanR1·Tg mice (line 111 and 241) on normal chow (NC) or a high-fat diet (HF) were analyzed by RT-PCR using a plasmid containing human and/or mouse AdipoR1 as control. n = 3 each (line 111), Control mice on a NC diet (n = 11); muscle-humanR1·Tg mice on a NC diet (n = 8). Control mice on a HF diet (n = 6); muscle-humanR1·Tg mice on a HF diet (n = 8) (line 241). (**c**) Protein levels of human and mouse AdipoR1 in skeletal muscle. Human and mouse AdipoR1 protein levels in skeletal muscle from muscle-humanR1·Tg mice (line 241) on a NC or a HF were analyzed by western blots using the antibody to detect human and mouse AdipoR1. Alpha-tubulin was used as a control for western blots to normalize the levels of AdipoR1. Control mice on a NC diet (n = 3); muscle-humanR1·Tg mice on a NC diet (n = 3). Control mice on a HF diet (n = 3); muscle-humanR1·Tg mice on a HF diet (n = 3) (line 241). (**d**) Protein levels of human and mouse AdipoR1·R2 in skeletal muscle. Human and mouse AdipoR1·R2 protein levels in skeletal muscle from AdipoR1-humanized mice on a HF were analyzed by western blots using the antibody to detect human and mouse AdipoR1·R2. Alpha-tubulin was used as a control for western blots to normalize the levels of AdipoR1·R2. Control mice on a HF diet (n = 3); R1·R2DKO mice on a HF diet (n = 3); AdipoR1-humanized mice on a HF diet (n = 3). All values are presented as means ± s.e.m. $^{**}P < 0.01$ compared to control mice (ANOVA followed by Tukey-Kramer multiple comparison tests).

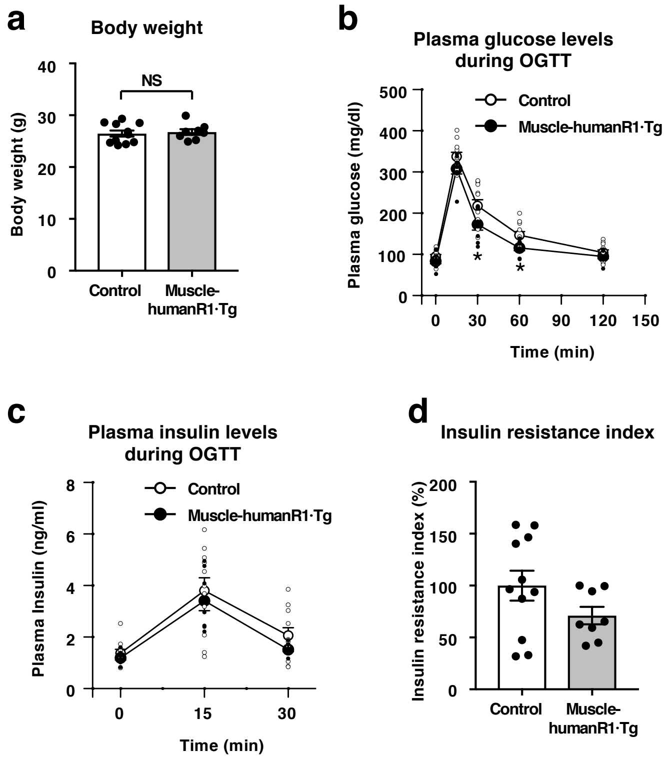

Supplementary Figure 2 | Increased insulin sensitivity in independent transgenic line 111 on a normal chow diet. Body weight (**a**), plasma glucose (**b**), plasma insulin (**c**) and insulin resistance index (**d**) during oral glucose tolerance test (OGTT) (1.5 g glucose per kg body weight) in control and muscle-humanR1·Tg mice (line 111) on a normal chow diet. All values are presented as means ± s.e.m. *$P < 0.05$ compared to control mice (unpaired two-tailed t-test). NS, not significant. Control mice: $n = 11$; muscle-humanR1·Tg mice: $n = 8$

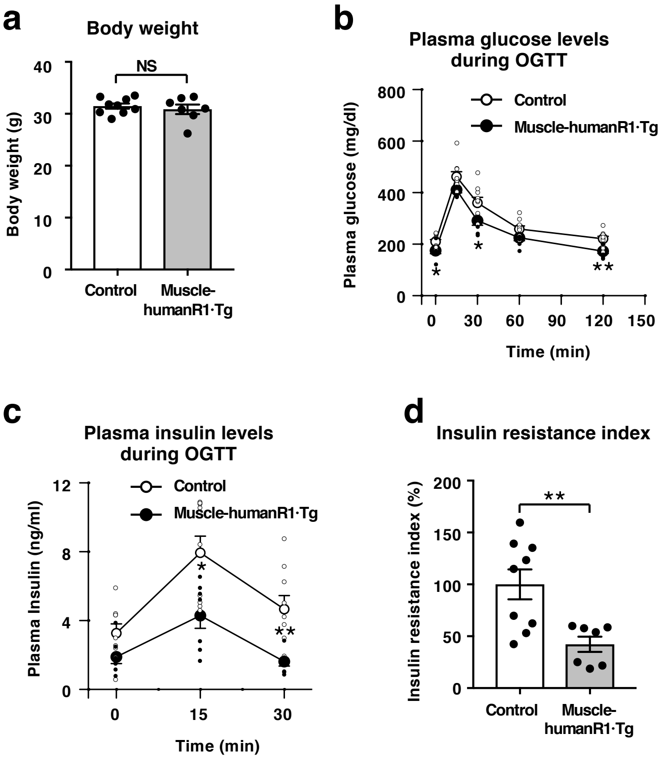

Supplementary Figure 3 | Increased insulin sensitivity in independent transgenic line 111 on a high-fat diet.

Body weight (**a**), plasma glucose (**b**), plasma insulin (**c**) and insulin resistance index (**d**) during oral glucose tolerance test (OGTT) (1.0 g glucose per kg body weight) in control and muscle-humanR1·Tg mice (line 111) on a high-fat diet. All values are presented as means ± s.e.m. *P < 0.05 and **P < 0.01 compared to control mice (unpaired two-tailed t-test). NS, not significant. Control mice: n = 9; muscle-humanR1·Tg mice: n = 7

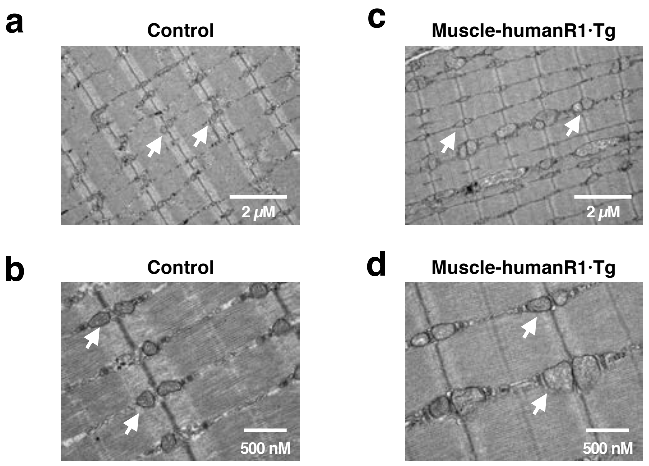

Supplementary Figure 4 | Mitochondrial morphology. Transmission electron microscope (TEM) images of skeletal muscle of control (**a**, **b**) and muscle-humanR1·Tg mice (**c** , **d**). TEM analysis of the mitochondria morphology in skeletal muscle of control and muscle-humanR1·Tg mice. Arrows point to examples of mitochondria in muscle are placed. **a**, **c**, scale bar = 2 μm; **b**, **d**, scale bar = 500 nm.

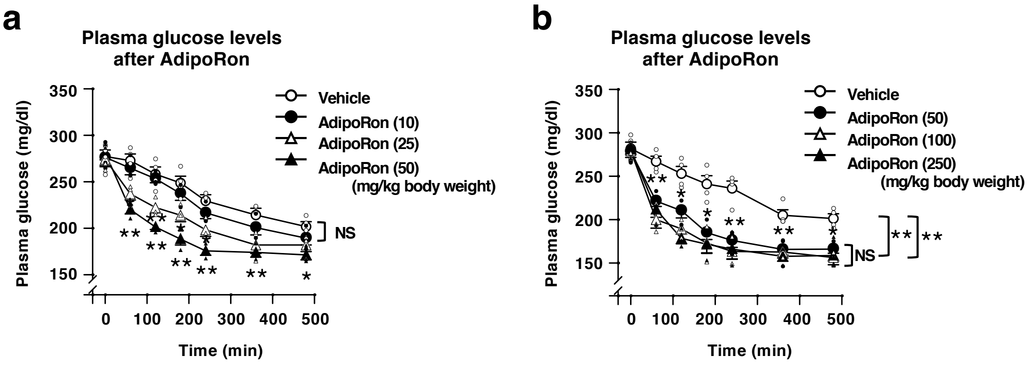

Supplementary Figure 5 | Glucose-lowering effects of AdipoRon on AdipoR1-humanized mice on a high-fat diet.

Plasma glucose levels in AdipoR1-humanized mice on a high-fat diet after oral administration of AdipoRon (10, 25, and 50 mg per kg body weight) (**a**) and AdipoRon (50, 100, and 250 mg per kg body weight) (**b**). All values are presented as means ± s.e.m. $*P < 0.05$ and $**P < 0.01$ compared to vehicle (ANOVA followed by Tukey-Kramer multiple comparison tests). NS, not significant. Vehicle, n = 4 each; AdipoRon (10, 25, 50, 100, and 250 mg per kg body weight), n = 3 each.

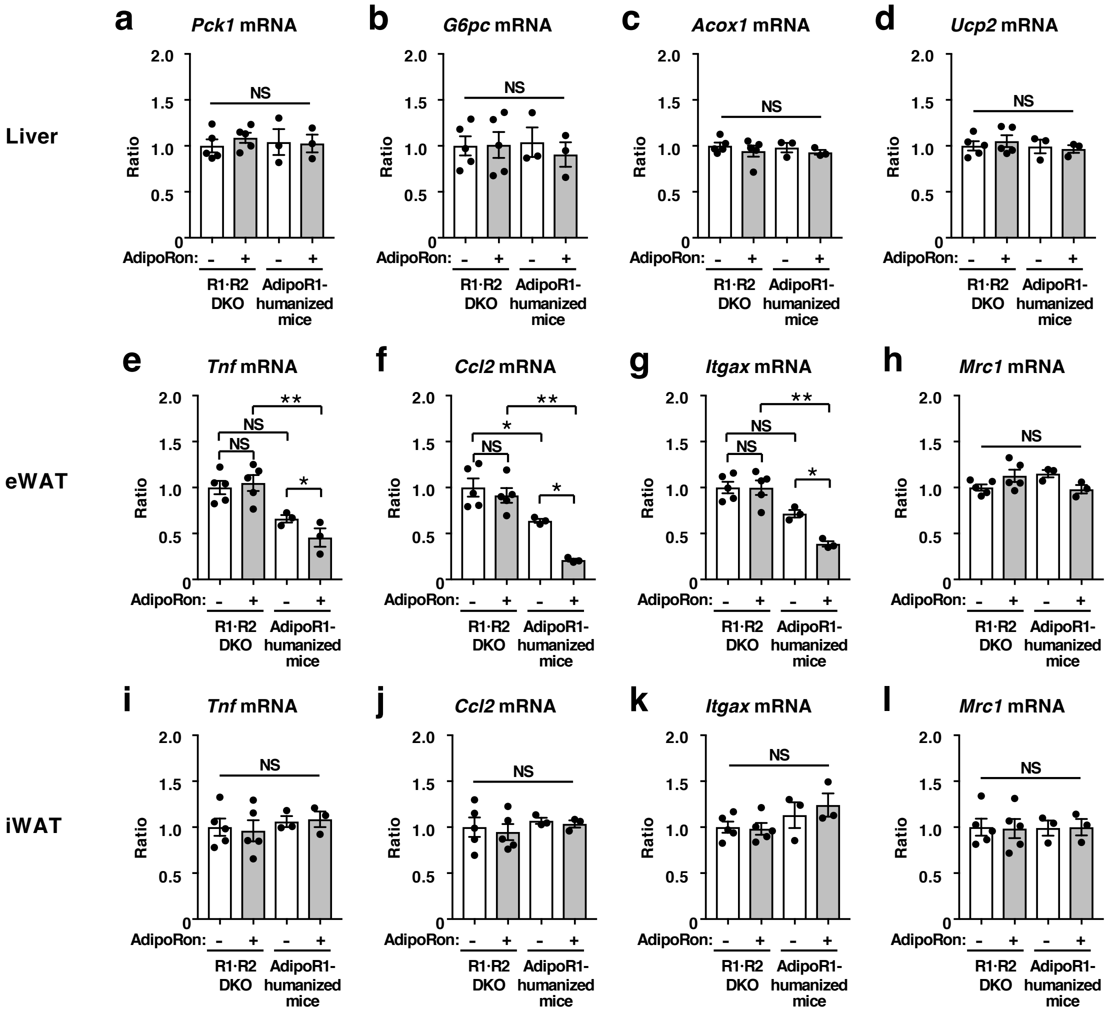

Supplementary Figure 6 | The effects of AdipoRon in liver and white adipose tissues of AdipoR-humanized mice on a high-fat diet.

Pck1 (**a**), *G6pc* (**b**), *Acox1* (**c**) and *Ucp2* (**d**) mRNA levels in liver of R1·R2DKO mice or AdipoR1-humanized mice on a high-fat diet. *Tnf* (**e**), *Ccl2* (**f**), *Itgax* (**g**) and *Mrc1* (**h**) mRNA levels in epididymal white adipose tissue (eWAT) of R1·R2DKO mice or AdipoR1-humanized mice on a high-fat diet. *Tnf* (**i**), *Ccl2* (**j**), *Itgax* (**k**) and *Mrc1* (**l**) mRNA levels in inguinal white adipose tissue of R1·R2DKO mice or AdipoR1-humanized mice on a high-fat diet. AdipoRon (50 mg per kg body weight) was orally administered once a day for 2 weeks. All values are presented as means ± s.e.m. $^*P < 0.05$ and $^{**}P < 0.01$ compared to control mice or as indicated (ANOVA followed by Tukey-Kramer multiple comparison tests). NS, not significant. R1·R2DKO mice (vehicle, n = 5; AdipoRon, n = 5), AdipoR1-humanized mice (vehicle, n = 3; AdipoRon, n = 3).

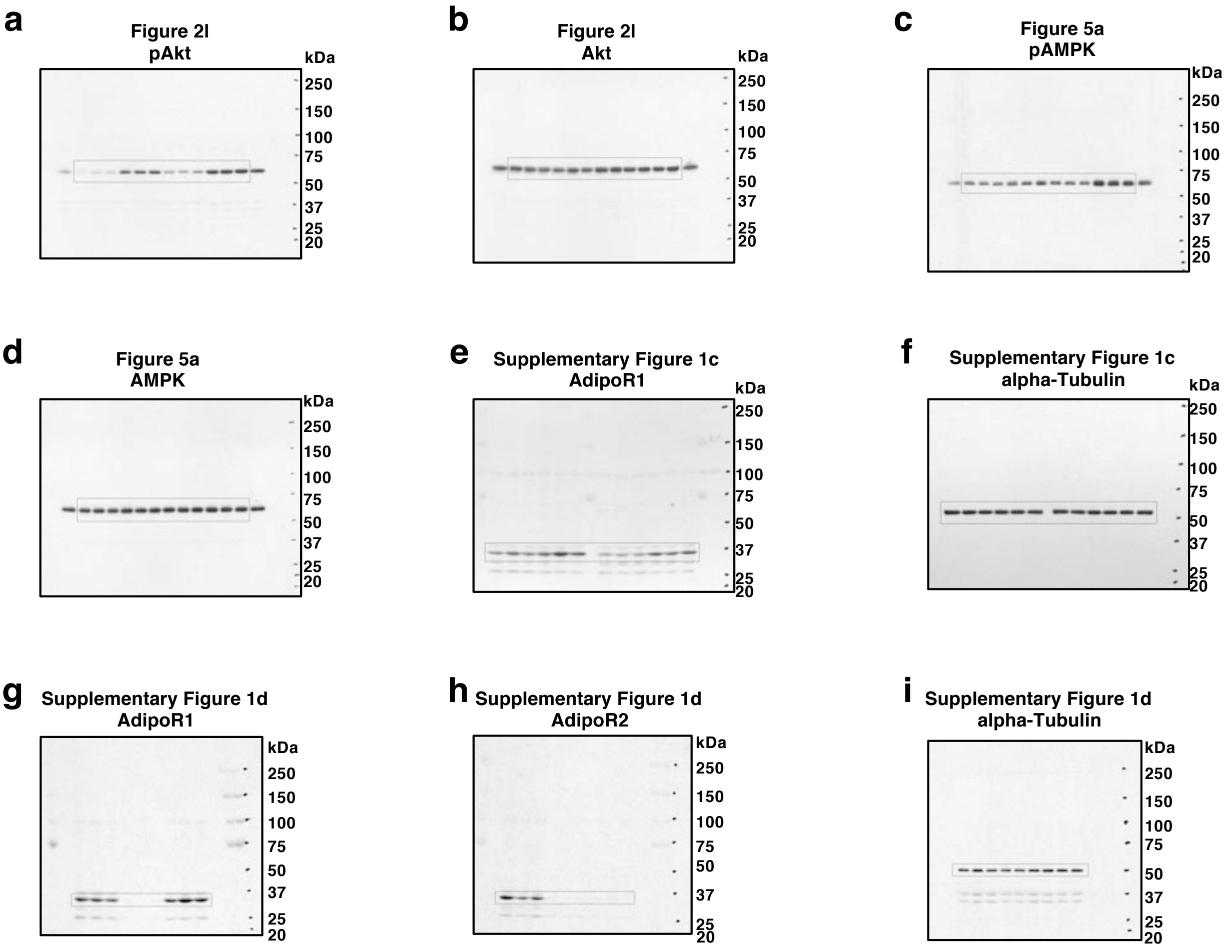

Supplementary Figure 7 | Raw western blots data.
Uncropped images of western blots in Figure 2l (**a**, **b**), Figure 5a (**c**, **d**), Supplementary Figure 1c (**e**, **f**) and Supplementary Figure 1d (**g**, **h**, **i**).
