# Supplementary material for: AdipoR agonist increases insulin sensitivity and exercise endurance in AdipoR-humanized mice
Source: Commun Biol. 2021 Jan 8;4:45. doi: 10.1038/s42003-020-01579-9 (PMC7794315; doi:10.1038/s42003-020-01579-9)
Supplement: Supplementary file 2 — Description of Additional Supplementary Files [file 42003_2020_1579_MOESM2_ESM.pdf]

Description of Additional Supplementary Files

File Name: Supplementary Data 1
Description: **The source data for the figures.**
The source data for Figure 1-5, Supplementary Figures 1-3, 4 and 5 are shown in Supplementary Data 1.
